# Kindergarten development and fertility intention: an empirical study from China

**DOI:** 10.3389/fsoc.2026.1617367

**Published:** 2026-02-16

**Authors:** Jingyi Wang, Lifei Gao, Guojun Wang

**Affiliations:** 1 School of Modern Service Management, Shandong Youth University of Political Science, Shandong, China; 2 School of Economics, Beijing Technology and Business University, Beijing, China; 3 School of Insurance and Economics, University of International Business and Economics, Beijing, China

**Keywords:** career aspiration, fertility intention, kindergarten development, leisure accessibility, work–family conflict

## Abstract

**Introduction:**

Faced with the inevitable trend of low fertility, how to build a more sustainable society that enables people to have the number of children they desire is becoming a global problem.

**Methods:**

Based on data from the China General Social Survey and China Education Statistics, we use Poisson regression, ordered logit regression, bootstrap analysis and other methods to explore the relationship between kindergarten development and fertility intention.

**Results:**

There is a positive correlation between developing kindergartens and fertility intention. The robustness tests prove the reliability of the research results. Developing kindergartens improves fertility intention by supporting career aspirations and increasing leisure accessibility. Women with a higher education level exhibit a stronger association with the development of kindergartens.

**Discussion:**

Although the empirical evidence is based on fertility intentions rather than realized fertility outcomes, the observed evidence may offer potential directions for optimizing demographic structures and fostering sustainable socioeconomic development. In the future, countries with low fertility should take measures to increase the supply of high-quality kindergartens and relieve childcare pressure to effectively solve the problem of low fertility.

## 1 Introduction

The total fertility rate (TFR) is a measure of the level of fertility over time. Population stability can be preserved above a TFR of 2.1 (United Nations Population Fund, 2023). Affected by rising female educational attainment, advancing urbanization, greater accessibility to contraception, reproductive health services, and reduced child mortality, fertility continue to decline (Goujon, 2025). According to the World Bank, many nations have already seen a decline in overall fertility rates below 2.1 by 2022 (World Bank, 2022). The overall fertility rate in Europe in 2022 was below 2.0, and the United States has remained at 1.7. Korea has the world’s lowest fertility rate, with an average of 0.8 children per woman, and Japan had an overall fertility rate of 1.3. Longitudinally, most developed western countries were already facing a relatively severe population crisis by the middle or end of the last century. In 2000, the number of countries which had a TFR below the replacement level of 2.1 in the world was around 70. In 2021, this number increased to nearly 110, of which nearly 40 countries and territories have experienced negative or zero population growth (Wu, 2020). The population decline and aging caused by low fertility present serious challenges to public finances, social security, pension systems, and health care, directly affecting a country’s socioeconomic development and national security (Harper, 2014).

To address the issue of declining fertility rates, governments worldwide have explored a range of family policies aimed at encouraging childbirth, which can be broadly categorized into three types: economic incentives, public service support, and cultural interventions (Gauthier, 2007). Economic incentives are the most direct approaches to promoting fertility, typically taking the form of cash subsidies, tax reductions, or housing support (Zhang et al., 2023). These measures have been shown to be more effective among low-income families, while their impact on high-income households tends to be weaker (Kalwij, 2010). Cultural intervention policies are designed to enhance fertility intentions by shifting societal norms. However, the effects of these policies are typically slow to materialize and difficult to quantify, as they often depend on the long-term construction of social consensus (Mcdonald, 2006). Public service support policies primarily include childcare services, parental leave systems, and educational subsidies. These measures have proven effective in increasing fertility intentions (Luci-Greulich and Thévenon, 2013).

While well-designed family support policies can moderately raise fertility rates or prevent them from declining further, such impacts are usually modest and far from restoring fertility to the replacement level (Gauthier and Gietel-Basten, 2025). According to the theory of demographic transition, low fertility is an inevitable outcome of socioeconomic development. At present, most regions of the world have undergone demographic transition, and more regions will experience fertility decline driven by educational advancement, urbanization, and improved child survival rates in the future. Given the inevitability of low fertility rates, the focus of policies should shift from efforts to raise fertility to a specific level toward building a fairer, more sustainable, and more caring society—one that enables people to have the number of children they desire [United Nations Population Fund (UNFPA), 2025]. As a key component of such a society, the enhancement of both the accessibility and quality of childcare services is strongly positively associated with the overall fertility trend (Bergsvik et al., 2021). Accordingly, this study integrates individual-level data from the Chinese General Social Survey (CGSS) database with provincial-level indicators from the China Education Database, to investigate the relationship between regional kindergarten development and individual fertility intentions (expected number of children), with a focus on elucidating the underlying mechanisms. The regional kindergarten development is a comprehensive concept in this study, including the number of kindergartens as well as the staffing of teachers and allocation of facilities.

Compared to prior studies, this research makes three significant contributions. First, to our knowledge, it provides the first empirical evidence linking comprehensive kindergarten development to fertility intentions. Unlike previous research that has focused on isolated factors such as cash subsidies or childcare quantity, this study offers a more holistic perspective, thereby complementing existing research on the determinants of fertility behavior. Second, it documents two key pathways—career aspiration and leisure accessibility—that help explain the correspondence between kindergarten development and fertility decisions. Third, the results offer actionable policy insights. Specifically, expanding kindergarten infrastructure and optimizing early childcare services emerge as viable strategies to address low fertility intentions. These findings provide critical theoretical and practical guidance for China to solve its low-fertility problem and achieve sustainable socioeconomic development.

The remainder of this paper is organized as follows. Section 2 introduces the decline in China’s fertility rate and the policy responses. Section 3 reviews the relevant literature and formulates theoretical hypotheses. Section 4 describes the data and variables. Section 5 introduces the methodology. The results are presented in Section 6. The final section discusses the results and provides conclusions.

## 2 Background: fertility decline and policy responses in China

As the largest developing economy, China is also faced with the issue of declining fertility rates. China’s TFR was approximately 6 in 1970, but it had declined significantly to around 1 by 2023, significantly below the replacement level of 2.1. The rapid decline in China’s fertility rate can be attributed to historical factors. In the early years of the People’s Republic of China, high fertility intentions among the population, coupled with relatively lenient government policies on childbirth, led to rapid population growth (National Bureau of Statistics of China, 2024). In response to the challenges posed by overpopulation, the central government implemented the family planning policy in the 1970s, advocating for one child per couple. This policy effectively controlled population growth, with scholars estimating that it prevented the birth of 264–320 million people between 1972 and 2000 (Wan et al., 2020).

**Figure 1:**
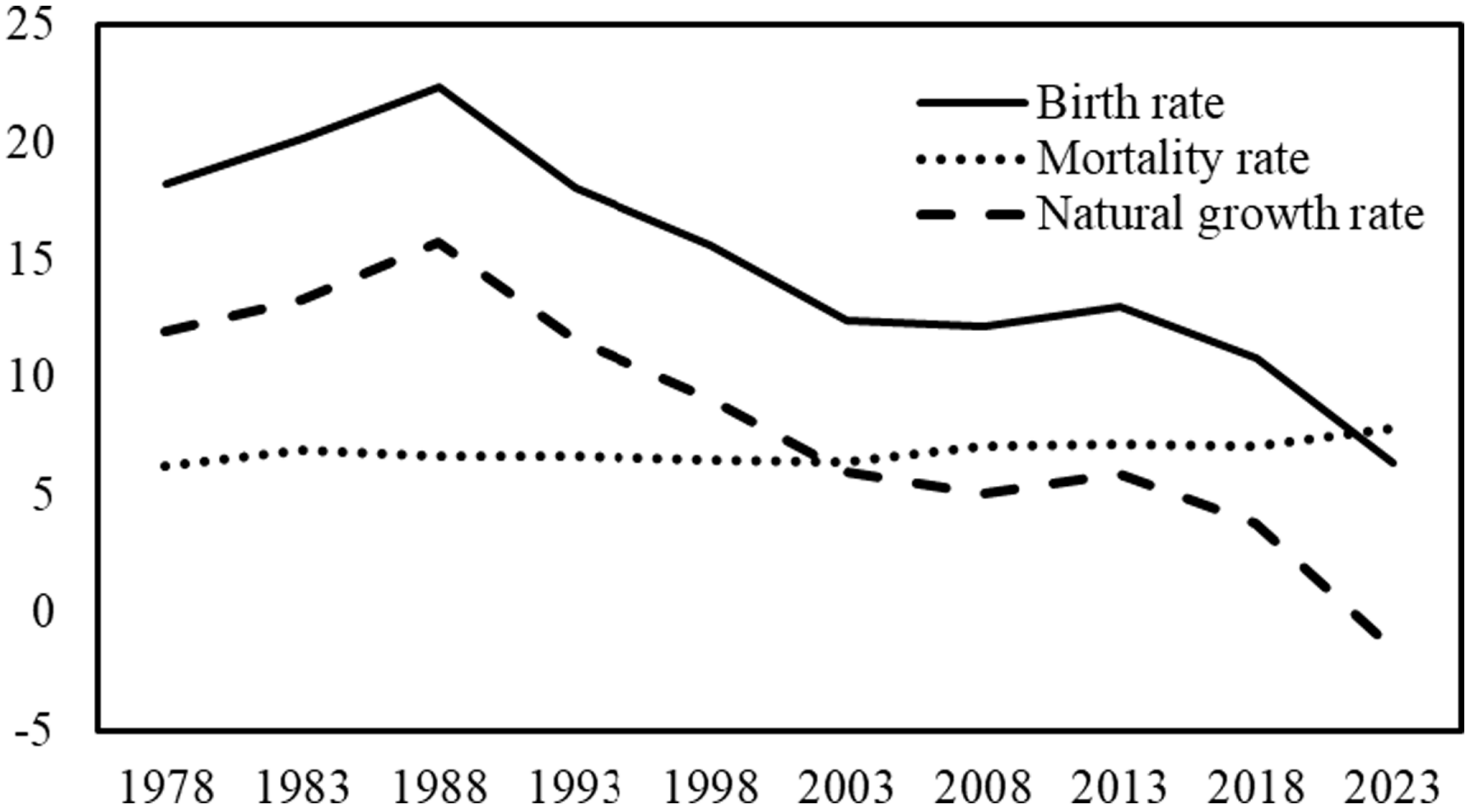
Changes in China’s birth, death, and natural growth rates, 1978–2023 (‰). Data source: National Bureau of Statistics of the People’s Republic of China.

Since the beginning of the 21st century, improvements in living standards and advancements in medical technology have led to increased life expectancy. However, the decline in fertility rates has directly contributed to the deepening of China’s population aging. Recognizing the severity of the population structure imbalance, the government began adjusting its family planning policies. In 2011, the two-child policy was introduced, and in 2021, the policy was further relaxed to allow couples to have three children. Nevertheless, due to the long-tail effects of the family planning policy and the high costs of child-rearing, the relaxation of birth restrictions has had limited effect in increasing fertility. Figure 1 illustrates that China’s birth rate has been declining steadily since 2017, reaching a record low in 2023 since 1949. The birth rate has fallen significantly below the death rate, resulting in a natural population growth rate of −1.48‰. With the intensification of population aging, if fertility rates remain persistently low, it will pose significant challenges to the sustainable development of the economy and society. Enhancing fertility rates is a crucial strategy for addressing the population crisis. Specifically, effectively increasing individuals’ fertility intentions represents a key leverage point for improving national fertility rates.

In response to national fertility policies, China has prioritized the development of childcare services. During 2016–2017, the government introduced policy documents to guide and incentivize social organizations to establish non-profit women’s and children’s hospitals, affordable nurseries, and kindergartens. Qualified kindergartens were further urged to provide public early education guidance for children aged 0–3 to parents and communities (The Central Committee of the Communist Party of China, & The State Council of the People’s Republic of China, 2015). In 2021, aligning with the three-child policy, China has promulgated policy documents that explicitly identify the establishment of an inclusive childcare service system as a key measure to enhance fertility support (The Central Committee of the Communist Party of China, & The State Council of the People’s Republic of China, 2021). By 2024, the State Council has released a report on childcare services, which highlights persistent challenges, including insufficient supply, uneven service quality, and inadequate policy support (The State Council of the People’s Republic of China, 2024). The report emphasized the urgent need to accelerate the expansion of childcare infrastructure and foster a socially supportive environment for childbearing to address these gaps.

## 3 Literature review and theoretical hypothesis

### 3.1 Childcare services and fertility intentions

Childcare for infants and young children has become a critical factor influencing family fertility intentions (Wang et al., 2019; Hong and Zhu, 2022). In China, inter-generational care plays a significant role in the childcare system, effectively alleviating parenting pressures for couples and enhancing fertility intentions to some extent (Gu et al., 2021). However, with demographic shifts and evolving societal attitudes, a noticeable trend toward the “de-familialization” of childcare has emerged, as an increasing number of young parents seek external childcare support. Survey results indicate that accessible childcare services rank as the top policy demand among individuals of childbearing age (Yang et al., 2021). Families urgently require affordable, state-supported childcare services to assist in child-rearing (Hong and Zhu, 2022). A study on the fertility intentions of women of childbearing age in Shanghai specifically highlights that the positive impact of grand-parental care on fertility intentions is significantly weaker than that of formal childcare services (Tian et al., 2020). Therefore, expanding the provision of public childcare services and offering high-quality social childcare support for infants and young children represent the most effective policies for boosting fertility intentions, particularly for second-child births.

Kindergartens are specialized institutions providing childcare services for young children. The development of these institutions is examined for its potential to enhance fertility intentions. A study conducted quasi-natural experiments to investigate the impact of kindergarten supply expansion and public childcare services on fertility intentions. Their findings reveal that policies promoting public kindergartens have a positive causal effect on family size, while public childcare services increase residents’ intentions to have a second child by approximately 16.2–20.5% (Jiang, 2021; Chen et al., 2023).

The positive impact of increased childcare services on fertility rates has been widely supported in developed countries. In Norway, a 20-percentage-point increase in childcare provision would result in no more than 0.05 additional children per woman in completed cohort fertility (Kravdal, 1996). It is also a Norwegian study that finds an increase in childcare coverage from 0 to 60% is associated with an average increase of 0.5–0.7 children among women up to 35 years old (Rindfuss and Choe, 2016). A major childcare reform in Germany resulting in a 10-percentage-point increase in public childcare coverage led to an additional 1.2 births per 1,000 women (Bauernschuster et al., 2016). Similarly, a positive effect of expanded childcare services on first birth odds also be observed in Belgium (Wood and Neels, 2019).

Based on the above analysis, the first hypothesis is proposed:

*H1*: The development of kindergartens can enhance individuals' fertility intentions.

### 3.2 Work–family conflict and fertility intentions

Work and family demands are inherently incompatible in terms of time, energy, and behavioral norms (Kahn et al., 1964). Work-related pressures can spill over into the family domain, and conversely, family-related demands can also affect work performance (Crouter, 1984). Following industrialization, significant changes in the nature and organization of work have exacerbated work–family conflicts (Brewster and Rindfuss, 2000). Women, in particular, have been disproportionately affected by these challenges (Connelly, 1992).

Work–family conflict is a significant factor contributing to low fertility intentions. Childbearing requires women to reallocate a portion of their time and energy from work to childcare. This reallocation often subjects them to severe time pressures in their professional lives and significantly reduces their fertility intentions (Begall and Mills, 2011). In more extreme cases, some women may forgo opportunities to re-enter the workforce due to childcare responsibilities. This inability to achieve self-fulfillment through their careers can further exacerbate the negative impact on their fertility intentions (Wesolowski, 2015). This issue is not limited to women. Men also experience tensions between career aspirations and childbearing, as they often perceive child-rearing as a potential disruption and source of pressure in their professional lives (Langdridge et al., 2005; Schytt et al., 2014). Additionally, reduced accessibility to formal early childhood education has been shown to decrease labor force participation rates and working hours (Doiron and Kalb, 2005; Viitanen, 2005). This, in turn, makes it difficult for individuals to achieve their career goals and exerts a negative influence on fertility intentions (Wesolowski, 2015).

Insufficient leisure time is another manifestation of work–family conflict. A primary source of work–family conflict lies in the allocation of time, where inadequate leisure time directly reflects this tension (Greenhaus and Beutell, 1985). Both the quantity and quality of leisure time can influence fertility decisions (Gimenez-Nadal and Sevilla, 2012). Moreover, the quality of leisure time, including its predictability and degree of autonomy, has a more significant impact on fertility intentions than its quantity (Craig and Mullan, 2011). Empirical studies have demonstrated that an increase of just 1 h of leisure time per week can significantly enhance fertility intentions (Gimenez-Nadal and Sevilla, 2012).

Career aspiration and leisure accessibility often compete with child-rearing responsibilities, which is a phenomenon observed in both women and men (Hakim, 2003; Martinez Mendiola and Cortina, 2024). Individuals can mitigate work–family conflicts arising from childbearing by seeking external support (Hobfoll, 1989). High-quality kindergartens typically provide superior childcare services, thereby alleviating the time and energy demands on parents. By entrusting their children to these institutions, parents are afforded greater opportunities to pursue career goals and engage in leisure activities, which in turn enhances their fertility intentions.

Based on the above analysis, the following hypotheses are proposed:

*H2a*: The development of kindergartens enhances fertility intentions by supporting parents' career aspirations.

*H2b*: The development of kindergartens enhances fertility intentions by increasing parents' leisure accessibility.

### 3.3 Theoretical framework: theory of planned behavior

In the Theory of Planned Behavior, a behavioral intention can find expression in behavior, which is influenced by the attitude toward the behavior, subjective norm and perceived behavioral control (Ajzen, 1991). The more favorable the attitude and subjective norm with respect to a behavior, and the greater the perceived behavioral control, the stronger should be an individual’s intention to perform the behavior under consideration. If a person can decide whether to perform the behavior, and intends to do so, he or she should be able to successfully translate that intention into action.

**Figure 2:**
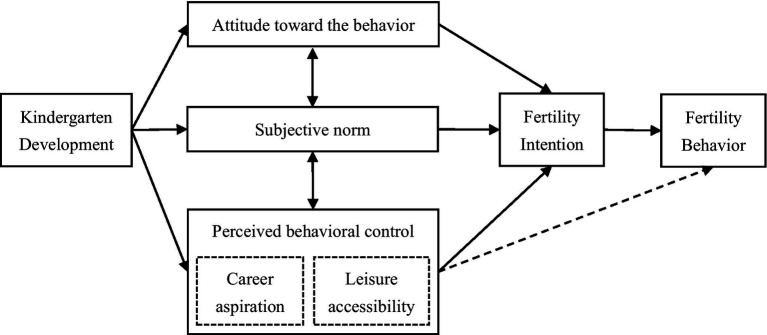
Theoretical framework based on the theory of planned behavior.

Building on the Theory of Planned Behavior (Ajzen, 1991), fertility intentions can capture the motivational factors that influence fertility behavior, which can be conceptualized as the result of three sets of determinants: (i) attitudes towards having (additional) children, (ii) subjective norms regarding appropriate family size, and (iii) perceived behavioral control, that is, the perceived ability to realize childbearing plans given time, financial and institutional constraints. In this framework, kindergarten development is expected to influence fertility intentions primarily through perceived behavioral control. By reducing time-intensive childcare demands and improving the compatibility between employment, leisure and parenting, high-quality kindergartens increase parents’ perceived ability to have (additional) children without sacrificing their career aspirations or leisure needs. At the same time, visible public investment in early childhood education may also foster more positive attitudes toward childbearing and contribute to a normative climate more supportive of larger family sizes. Thus, the Theory of Planned Behavior provides a coherent micro-foundation for our hypotheses: we expect higher levels of kindergarten development to be associated with stronger fertility intentions overall (H1), and to operate in particular through the mechanisms of career aspiration and leisure accessibility (H2a, H2b). Figure 2 illustrates the theoretical framework of this paper based on the Theory of Planned Behavior.

## 4 Data and variables

### 4.1 Data source

The data utilized in this study are sourced from the Chinese General Social Survey (CGSS) database, the China Family Panel Studies (CFPS) database and the China Education Database. Both the CGSS and the CFPS have surveyed respondents’ fertility intentions. However, the CGSS conducts such surveys in each survey year, while the CFPS only included this survey in 2018. To ensure the credibility of the research results, we use the CGSS for the baseline regression and the CFPS for the robustness test. China Education Database provides detailed information on the development of kindergartens in each province every year, including the number of kindergartens, kindergarten staff, and operational conditions. To make full use of the individual samples and explore the impact of kindergarten development on individual fertility intention, we match the data from the CGSS for the years 2012, 2013, 2015, 2017 and 2018 with provincial-level kindergarten indicators from China Education Database for the corresponding years. Considering that the fertility intentions among individuals outside the childbearing age range make a limited contribution to the increase in social fertility, we restrict our sample to individuals aged 16–49. After removing missing values and outliers, we construct a database comprising 20,434 samples.

### 4.2 Variables

#### 4.2.1 Dependent variables

The dependent variable is people’s fertility intention. Fertility intentions can be categorized into three levels: the ideal, expected, and intended number of children. These levels reflect varying degrees of alignment with actual fertility behavior. The ideal number of children reflects individuals’ attitudes toward childbearing and is not closely related to actual fertility behavior (Wu, 2020). The expected number represents the number of children individuals would like to have, incorporating their intrinsic needs. The intended number, which is most closely related to actual fertility behavior (Ryder and Westoff, 2017), reflects the number of children individuals plan to have, taking into account both realistic conditions and intrinsic needs. The CGSS questionnaire includes question related to fertility intentions: If there are no policy restrictions, how many children would you like to have? The question investigates individuals’ expected number of children, which reflects social demand for fertility (McClelland, 1983) and can be a useful measure of fertility intention.

#### 4.2.2 Independent variables

The independent variable in this study is kindergarten development, which is represented by multiple indicators in the China Education Database. To make the study more concise, we use principal component analysis (PCA) to reduce the dimensionality of these indicators and consolidate several basic indicators into a single comprehensive variable reflecting the overall level of kindergarten development in each province. Based on the classifications in the China Education Database, five basic indicators are used: the number of kindergartens, faculty levels, indoor areas, outdoor areas, and teaching resources. The indicator composition is detailed in Supplementary Table S1.

The 15 basic indicators have a Kaiser–Meyer–Olkin value of 0.877 and a Bartlett significance of 0.000, indicating they are highly correlated with each other and satisfy the conditions of PCA. Using a cumulative variance contribution of over 85% (Zhou et al., 2008), we extract three principal components, respectively, representing the facilities level, the faculty level and the number of kindergartens (see Supplementary Tables S2, S3). The final kindergarten variables are derived based on the relative weights of the variance contributions of each principal component to the cumulative variance of the extracted principal components.

#### 4.2.3 Control variables

According to previous studies, factors influencing fertility intentions can be categorized into individual, family, and social characteristics. Accordingly, sex (Seccombe, 1991), age (Berninger et al., 2011), self-rated-health (Boivin et al., 2018), education (Testa, 2014), household income and number of properties (Hanappi et al., 2017) are selected as control variables to account for individual characteristics. Marital status (Rijkin and Liefbroer, 2009; Vignoli et al., 2013) is selected as a control variable to account for family characteristics. Social insurance participation is selected as a control variable to account for social characteristics. Some scholars argue that social security can alleviate the financial pressure in old age, thereby reducing the desire to have children (Boldrin et al., 2015). In contrast, others suggest that the burden of social security contributions crowds out current consumption, thereby increasing fertility intentions (Huang and Xu, 2019). To account for national-specific conditions, ethnicity and household registration status are included as additional control variables. Specifically, individuals of Han ethnicity tend to have lower fertility intentions compared to other ethnic groups, and fertility rates are generally lower in urban areas (Chen and Shi, 2002).

#### 4.2.4 Mediating variables

##### 4.2.4.1 Career aspiration

The difficulty in realizing career aspirations is a significant factor negatively impacting fertility intentions (Wesolowski, 2015). People with higher socioeconomic status are more likely to actively explore career directions and persist in their goals, thereby maximizing the realization of their career aspirations (Hu et al., 2022). Since there is a significant positive relationship between socioeconomic status and career aspirations (Howard et al., 2011), this study employs the question, “Which social stratum do you believe you belong to?” to measure respondents’ self-socioeconomic status, serving as a proxy for career aspiration. Higher level of self-socioeconomic status indicates that an individual’s career aspirations are relatively well satisfied.

##### 4.2.4.2 Leisure accessibility

When childcare support is unavailable, individuals who prioritize leisure tend to exhibit lower fertility intentions (Arranz Becker and Lois, 2013). This study employs the question (i.e., “How often have you socialized/relaxed/studied in the past year?”) to measure respondents’ leisure engagement, which is used as a proxy for leisure accessibility. Frequent engagement in social activities, relaxation, and study suggests higher levels of leisure accessibility.

Table 1 is the definition of variables.

**Table 1:** Definition of variables.

Variables	Indicator	Definition
Dependent variable	Child	Expected number of children
Independent variable	Kindergarten	Kindergarten development level
Control variable	Gender	Male = 1; female = 0
	Age	Respondents’ age at the time of the survey
	Household registration	Agricultural = 1; non-agricultural = 0
	Ethnicity	Han = 1; minority = 0
	Marital status	Married = 1; unmarried = 0
	Education	Integer variables from 1 to 14; a high value indicates a high level of education
	Income	Logarithm of household income
	House	Number of properties
	Self-rated-health	Integer variables from 1 to 5; a high value indicates good health
	Basic medical insurance	Yes = 1; No = 0
	Basic retirement insurance	Yes = 1; No = 0
Mediating variable	Career aspiration	Integer variables from 1 to 10; a high value indicates a high degree of self-fulfillment
	Leisure accessibility	Integer variables from 3 to 15; a high value indicates more leisure engagement

## 5 Methodology

### 5.1 Poisson regression

The dependent variable is fertility intention, expressed by expected number of children, which are typically count variables. After testing, there is no overdispersion issue in the data. Therefore, the poisson regression model is used.


$$E(\text{chil}d_{i}|\text{kindergarte}n_{i},X_{i})=\alpha_{0}+\alpha_{1}\text{kindergarte}n_{i}+\alpha_{2}X_{i}+\varepsilon_{i}$$ (1)

Equation 1 is the expression for poisson regression, where $\text{chil}d_{i}$ denotes the number of children that individual i expects to have. $\text{kindergarte}n_{i}$ indicates the development of kindergartens in each province. $X_{i}$ indicates a range of control variables, including sex, age, household registration, ethnicity, marital status, education, income, house, self-rated-health, social security participation. We also control for the impact of time. $\;\varepsilon_{i}$ is the residual term of the model. Standard errors are clustered on the provincial level.

### 5.2 Ordered logit regression

In addition, we use ordered logit regression to ensure the robustness of the results.


$$\begin{array}{c} \text{logit}(P(\text{chil}d_{i}\leq i|\text{kindergarte}n_{i},X_{i}))=\alpha_{0}+\alpha_{1}\text{kindergarte}n_{i}\\ +\alpha_{2}X_{i}+\varepsilon_{i} \end{array}$$ (2)

Equation 2 is the expression for ordered logit regression. The meaning of each variable is consistent with that in the Poisson Regression.

### 5.3 Bootstrap analysis

We utilize the Bootstrap method for mediating association analysis to explore the pathways that help explain the relationship between kindergarten development and fertility intentions. This statistical approach generates confidence intervals through repeated sampling procedures, does not rely on strict normality assumptions, and is applied to evaluate the statistical significance of the indirect linkage between an independent variable and a dependent variable that operates through a mediating variable.

## 6 Results

### 6.1 Descriptive statistics

The kindergarten variable constructed by PCA is a continuous variable with a mean of 0. Provinces with a value greater than 0 are considered to have higher levels of kindergarten development compared to the national average, while those with a value less than 0 are considered to have lower levels. Based on this criterion, the sample is divided into two groups: a low kindergarten development group and a high kindergarten development group. Table 2 presents the distribution of fertility intentions across different subsamples.

**Table 2:** The distribution of fertility intentions across different subsamples.

Variable	*N*	Child		
		Mean	Min	Max
Kindergarten				
High level	9,201	1.99	0	21
Low level	11,233	1.83	0	21
Gender				
Male	9,616	1.92	0	21
Female	10,818	1.89	0	12
Age				
16–20	800	1.73	0	10
20–30	4,949	1.85	0	21
30–40	6,768	1.89	0	21
40–49	7,917	1.96	0	12
Household registration				
Non-agricultural	7,392	1.79	0	11
Agricultural	13,042	1.97	0	21
Nation				
Han	18,733	1.89	0	21
Minority	1701	2.09	0	10
Marital status				
Married	16,268	1.94	0	21
Unmarried	4,166	1.76	0	21
Education				
Junior High School	10,883	1.98	0	21
Senior High School	2,758	1.85	0	11
College	6,453	1.80	0	10
Graduate student	340	1.84	0	6
Income				
High level	10,796	1.84	0	11
Low level	9,638	1.97	0	21
House				
0	1807	1.80	0	10
1	15,426	1.91	0	21
2 or more	3,201	1.93	0	11
Self-rated-health				
1	296	2.03	0	10
2	1,494	2.01	0	21
3	3,675	1.92	0	10
4	8,592	1.87	0	12
5	6,377	1.90	0	21
Basic medical insurance				
Yes	18,368	1.91	0	21
No	2066	1.83	0	11
Basic retirement insurance				
Yes	12,710	1.90	0	12
No	7,724	1.91	0	21
Total	20,434	1.90	0	21

As shown in Table 2, the expected number of children for the entire sample lies between that of the low and high kindergarten development groups. Specifically, the high kindergarten development group exhibits significantly higher fertility intentions compared to the low kindergarten development group, with the number of expected children being significantly higher in the high kindergarten development group. Based on these preliminary findings, we can tentatively infer that higher levels of kindergarten development may be associated with increased fertility intentions. However, given that fertility intentions are shaped by a multitude of factors, further empirical analysis is required to confirm whether there is a significant relationship between kindergarten development and fertility intentions.

### 6.2 Baseline regression results

Table 3 shows the specific impact of kindergartens on fertility intention. Columns (1)–(2) present the relationship between kindergarten development and the expected number of children derived from poisson regression. Columns (3)–(4) show the results obtained through ordered logit regression. The results indicate that enhancing kindergarten development is significantly associated with increased fertility intentions. Poisson regression indicates that each one-point increase in the total kindergarten score is associated with a 9% increase in the expected number of children. Ordered logit regression indicates that for each one-point increase in the quality level of kindergartens, the odds of having a higher level of fertility intention are 1.9 times the original.

**Table 3:** Kindergarten development and fertility intention.

Variables	Child			
	(1)	(2)	(3)	(4)
Kindergarten	1.061*** (3.01)	1.095*** (3.74)	1.503*** (3.44)	1.889*** (4.11)
Control	No	Yes	No	Yes
Provincial cluster	Yes	Yes	Yes	Yes
Obs	20,434	20,434	20,434	20,434
R-squared	0.001	0.005	0.009	0.034

*T* statistic value in parentheses. ***p < 0.01, **p < 0.05, *p < 0.1.

Columns (1)–(2) are incidence rate ratios of Poisson regression; columns (3)–(4) are odds ratios of ordered logit regression.

### 6.3 Endogeneity tests

To solve potential endogeneity problems arising from reverse causality, omitted variables, and measurement error, we employ instrumental variables to mitigate the impact of these problems on the estimation results.

We adopt the frequency of the term “kindergarten” mentioned in provincial educational development plans, which are released every 5 years, as an instrumental variable for kindergarten development. Theoretically, the frequency of the term “kindergarten” mentioned in provincial plans is indicative of the priority given to kindergarten development. A higher frequency of mentions indicates greater attention to and better implementation of kindergarten development within the province. Moreover, how kindergartens are referred to in policy documents does not directly influence individuals’ fertility intentions. This characteristic satisfies the criteria for selecting instrumental variables. To further mitigate the potential influence of contemporaneous policies on fertility intentions, we employ lagged mentions as instrumental variables. Specifically, the frequency of the term “kindergarten” mentioned in plans from 2016 to 2020 serves as the instrumental variable for the years 2012, 2013, and 2015, while mentions from 2021 to 2025 plans are used for the analysis of 2017 and 2018 (Reed, 2015).

Table 4 presents the regression results. As shown, when the frequency of kindergarten mentioned (kindergarten_f) is employed as the instrumental variable, the coefficients in the first-stage regression are significantly positive. The LM statistic of 8.38 and the Wald F statistic of 24.14 indicate that the instrumental variables are effective. The results of the second-stage regression show that there is still a significant positive relationship between kindergarten development and fertility intention after using the instrumental variable. This is consistent with the findings of the basic regression.

**Table 4:** Kindergarten development and fertility intention: IV regression.

Variables	First stage	Second stage
	Kindergarten	Child
Kindergarten		0.275*** (5.50)
Kindergarten-f	0.028*** (5.31)	
Control	Yes	Yes
LM statistic	8.38	
Wald *F* statistic	24.14	
Obs	19,492	

*T* statistic value in parentheses. ***p < 0.01, **p < 0.05, *p < 0.1.

The reduction in observations is attributable to the absence of educational development plans in some provinces.

### 6.4 Robustness tests

#### 6.4.1 Replace the database

The China Family Panel Studies (CFPS) 2018 also investigates individuals’ fertility intention. The indicator construction, variable selection, and empirical methodology are consistent with those used in the basic regression. Table 5 illustrates that an increase in the level of kindergarten development is associated with a significant rise in the expected number of children.

**Table 5:** Kindergarten development and fertility intention: Alternative the database.

Variables	Child			
	(1)	(2)	(3)	(4)
Kindergarten	1.028*** (3.14)	1.024*** (2.96)	1.246*** (3.88)	1.220*** (3.56)
Control	No	Yes	No	Yes
Provincial cluster	Yes	Yes	Yes	Yes
Obs	12,638	12,638	12,638	12,638
R-squared	0.003	0.008	0.032	0.075

*T* statistic value in parentheses. ***p < 0.01, **p < 0.05, *p < 0.1.

Columns (1)–(2) are incidence rate ratios of Poisson regression; columns (3)–(4) are odds ratios of ordered logit regression.

#### 6.4.2 Replace the dependent variable

A higher proportion of multiple-member households in a province indicates a greater prevalence of families with higher fertility intentions. Based on the China Statistical Yearbook, we use the proportion of households with four or more members, five or more members, six or more members in each province as proxies for the dependent variable in the basic regression analysis. Table 6 illustrates that an increase in the level of kindergarten development is significantly associated with a higher proportion of multiple-member households.

**Table 6:** Kindergarten development and fertility intention: alternative the dependent variable.

Variables	Household of 4 or more		Household of 5 or more		Household of 6 or more	
	(1)	(2)	(3)	(4)	(5)	(6)
Kindergarten	0.068^***^ (4.24)	0.095^***^ (4.50)	0.040^***^ (4.50)	0.054*** (4.37)	0.025^***^ (4.68)	0.032^***^ (4.19)
Control	No	Yes	No	Yes	No	Yes
Provincial cluster	Yes	Yes	Yes	Yes	Yes	Yes
Obs	20,434	20,434	20,434	20,434	20,434	20,434
R-squared	0.255	0.470	0.264	0.448	0.303	0.436

*T* statistic value in parentheses. ***p < 0.01, **p < 0.05, *p < 0.1.

#### 6.4.3 Add policy control variables

The national fertility regulation policy may affect residents’ fertility intention (Lan, 2021). To mitigate the potential impact of fertility policies on our study, we include dummy variables for the two-child and three-child policies in China as control variables. Table 7 illustrates that the regression results are consistent with those of the basic regression analysis.

**Table 7:** Kindergarten development and fertility intention: add policy control variables.

Variables	Child			
	(1)	(2)	(3)	(4)
Kindergarten	1.095*** (3.74)	1.095*** (3.74)	1.889*** (4.11)	1.889*** (4.11)
Control	Yes	Yes	Yes	Yes
Provincial cluster	Yes	Yes	Yes	Yes
Policy	No	Yes	No	Yes
Obs	20,434	20,434	20,434	20,434
R-squared	0.005	0.005	0.034	0.034

*T* statistic value in parentheses. ***p < 0.01, **p < 0.05, *p < 0.1.

Columns (1)–(2) are incidence rate ratios of Poisson regression; columns (3)–(4) are odds ratios of ordered logit regression.

### 6.5 Mechanism analysis

**Figure 3:**
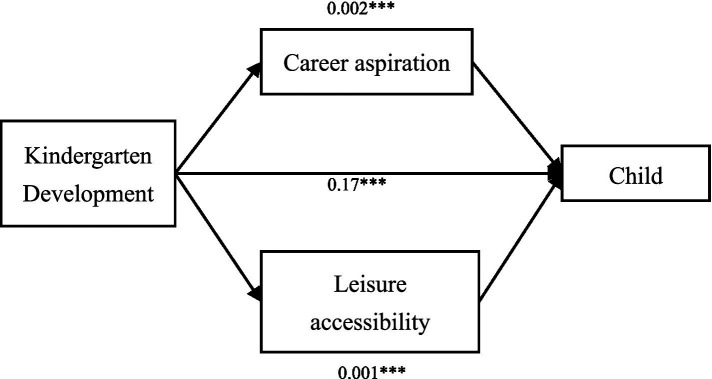
Path diagram of the mechanism analysis for the association between kindergarten development level and fertility intention. ***p < 0.01, **p < 0.05, *p < 0.1.

We employ levels of self-fulfillment and frequency of social activities, relaxation, and study as proxies for the career aspiration and leisure accessibility, respectively. Using Bootstrap analysis, the linkages between kindergarten development and fertility intentions are explored. Figure 3 illustrates that career aspiration and leisure accessibility partially account for the association between kindergarten development and fertility intentions.

### 6.6 Heterogeneity analysis

Given that women generally face greater work–family conflicts than men (Connelly, 1992), this section focuses on women, with particular attention to the relationship between kindergarten development and the fertility intentions of women with different educational backgrounds and household registration statuses. Specifically, female samples are divided into four groups: urban women with low education, urban women with high education, rural women with high education, and rural women with low education. The association between kindergarten development and fertility intention is then examined across these groups. Table 8 presents the regression results.

**Table 8:** Kindergarten development and fertility intention: heterogeneity analysis.

Group	Child	
	(1)	(2)
(1)	0.986 (−0.12)	0.914 (−0.09)
(2)	1.099*** (4.87)	1.829*** (5.10)
(3)	1.101*** (3.30)	2.008*** (3.39)
(4)	1.028 (0.68)	1.228 (0.73)

*T* statistic value in parentheses. ***p < 0.01, **p < 0.05, *p < 0.1.

Columns (1) are incidence rate ratios of Poisson regression; columns (2) are odds ratios of ordered logit regression.

Groups (1)–(4) are urban women with low education, urban women with high education, rural women with high education, and rural women with low education, respectively.

The results indicate that improved kindergarten development is significantly positively associated with fertility intention among women with higher educational levels. Poisson regression results show that each one-point increase in the total kindergarten score corresponds with an approximate 10% increase in the expected number of children. Ordered logit regression results reveal that for each one-point increase in the quality level of kindergartens, the odds of reporting a higher level of fertility intention are approximately twice the baseline value. In contrast, for women with lower educational levels, the linkage between kindergarten quality and fertility intention is not statistically significant.

## 7 Discussion

This study analyzes the relationship between kindergarten development levels and fertility intentions using Poisson regression models based on five waves of CGSS data and the China Education Database. The results indicate that higher levels of kindergarten development are positively associated with increased fertility intentions. Each one-point increase in the total kindergarten score is associated with a 9% increase in the expected number of children. Hypothesis 1 is validated. Our findings are consistent with the previous research (Jiang, 2021; Chen et al., 2023). However, prior studies operated on markedly different time horizons, which range from short-term annual birth rates to long-term completed fertility, and are therefore not directly comparable in effect size or interpretation. And these studies primarily examined the relationship between kindergarten supply and fertility intentions from the perspectives of kindergarten quantity and availability, without focusing on the overall development level of kindergartens. It is worth noting that although the estimated percentage change in fertility intentions appears substantial, the corresponding absolute increase is relatively modest. This discrepancy stems from the low baseline value of expected number of children, which serves as the explained variable in our research. Therefore, while kindergarten development is statistically significantly associated with higher fertility intention, its practical relevance to the overall fertility level is limited and should not be overstated. Additionally, the potential directional nature of the relationship between fertility intentions and kindergarten development requires attention. For instance, regions with higher fertility intentions tend to have more children, which may correspond with greater investment in kindergarten construction in these regions. To address potential endogeneity issues, we employ instrumental variables, and the regression results remain consistent with the baseline findings. Robustness checks including sample adjustments, dataset replacements, and changes in dependent variables, as well as the consideration of policy implications, confirmed the stability of the baseline regression results.

In contexts with low-quality childcare services, the negative relationship between work-family imbalance-induced time pressures and fertility intentions becomes more pronounced (Begall and Mills, 2011). This suggests that higher quality of childcare services is associated with a reduction in the negative linkage between work-family imbalance and fertility intentions. We utilize the Bootstrap method to examine the mechanisms that help explain the linkage between kindergarten development and fertility intentions. Our study provides preliminary evidence suggesting that career aspiration and leisure accessibility may act as potential mediating factors in the relationship linking kindergarten development to fertility intentions. In contexts with higher kindergarten development levels, we observe less parental involvement in time-intensive childcare, alongside greater time allocated to career goals and leisure activities among parents. Hypothesis 2a and Hypothesis 2b are also validated. It should be noted that data constraints have limited our options in selecting proxies for mediating variables. While self-socioeconomic status can reflect the degree of career aspiration attainment to a certain extent, it is more indicative of attitudinal dimensions and neglects the impact of behavioral engagement on the realization of occupational aspirations. Moreover, access to leisure is not confined to social interaction, learning, and rest alone. Although our findings cannot fully capture the pathways through which kindergarten development is associated with fertility intentions, they can to some extent illustrate the linkages underlying this relationship.

The association between kindergarten development and fertility intentions varies across different demographic groups, which reflects the documented linkage between kindergarten availability and the mitigation of work–family conflicts, as well as corresponding trends in fertility intentions. Our findings indicate that compared to women with lower education levels, kindergarten development exhibits a stronger positive correspondence with fertility intentions among women with higher education levels. Compared to individuals with lower education levels, highly educated individuals often face more demanding and rapid lifestyles, making it challenging to balance household responsibilities and professional work (Schieman and Glavin, 2011; Rubenstein et al., 2022). High-quality kindergartens provide superior childcare services, a characteristic that corresponds with better work-family balance among parents. Therefore, these groups exhibit greater sensitivity to variations in kindergarten development levels.

The findings that high-quality kindergartens are associated with improved fertility intentions provide preliminary insights for addressing low-fertility challenges, though interpretations and applications of these insights should be cautious. This is because the empirical evidence is based on fertility intentions rather than realized fertility outcomes—with the translation of intentions into actual fertility behavior still requiring further verification. Nevertheless, the observed evidence may offer potential directions for optimizing demographic structures and fostering sustainable socioeconomic development in low-fertility countries, especially those where parents face more severe work–family conflicts. First, governments may warrant recognizing the potential role of kindergarten development in policies aimed at shaping fertility intentions and could consider proactive measures to expand the supply of kindergartens, enhance their accessibility, and thereby alleviate the childcare burden on families. Second, policymakers might focus on improving the quality of kindergarten services as a potential means to support fertility intentions. Feasible measures could include strengthening kindergarten teacher training, optimizing the allocation of educational resources, and upgrading supporting infrastructure. Finally, governments could explore encouraging and supporting eligible organizations and enterprises to establish on-site kindergartens and other welfare-oriented childcare facilities. Promoting social participation in the provision of kindergarten services may also help build a network of reliable, affordable, and accessible childcare resources.

There are still some limitations to this study. First, the price of kindergarten services, pedagogical quality, staff-child ratios, or parental satisfaction are important indicators for measuring the development of kindergartens. However, due to data constraints, we are unable to integrate these variables into the comprehensive kindergarten development index or investigate the characteristics of their linkage with fertility intentions. Second, the observed association between kindergarten development and fertility intentions may be partially moderated by family support, where reduced access to informal extended family childcare is associated with increased reliance on formal childcare provisions. Limited by data availability, we are unable to control for factors such as family support in the model. Third, with respect to the mechanisms that underpin the correspondence, the two indirect linkages identified in the study account for a relatively small share of the overall relationship, indicating limited explanatory power. Therefore, the specific pathways that characterize the association between kindergarten development and fertility intentions require further empirical investigation.

## Data Availability

The original contributions presented in the study are included in the article/Supplementary material, further inquiries can be directed to the corresponding author.
