# Supplementary material for: Kindergarten development and fertility intention: an empirical study from China
Source: Front Sociol. 2026 Feb 16;11:1617367. doi: 10.3389/fsoc.2026.1617367 (PMC12950552; doi:10.3389/fsoc.2026.1617367)
Supplement: Supplementary file 1 [file Table_1.DOCX]

**Supplementary materials**

**Table S1**

*Indicator Composition of Kindergarten Development*

| **Variables** | **Sub indicators** | **Basic indicators** | **Meaning of the basic indicators** |
| --- | --- | --- | --- |
| Kindergarten | Number of kindergartens | Number of kindergartens | Number of kindergartens per 10,000 people |
|  |  | Number of kindergarten classes | Number of kindergarten classes per 10,000 people |
|  | Faculty levels | Number of kindergarten directors | Number of kindergarten directors per 10,000 people |
|  |  | Number of teachers | Number of teachers per 10,000 people |
|  |  | Number of health practitioners | Number of health practitioners per 10,000 people |
|  |  | Number of caregivers | Number of caregivers per 10,000 people |
|  | Indoor areas | Activity room area | Activity room area as a proportion of residential land area（%） |
|  |  | Library area | Library area as a proportion of residential land area（%） |
|  |  | Sleeping room area | Sleeping room area as a proportion of residential land area（%） |
|  |  | Health room area | Health room area as a proportion of residential land area（%） |
|  |  | Toilets area | Toilets area as a proportion of residential land area（%） |
|  |  | Kitchen area | Kitchen area as a proportion of residential land area（%） |
|  | Outdoor areas | Green spaces area | Green spaces area as a proportion of residential land area（%） |
|  |  | Sports fields area | Sports fields area as a proportion of residential land area (%) |
|  | Teaching resources | Number of books | Number of books per 10,000 people |

**Table S2**

*Contribution of Variance of Each Principal Component*

| **Factor** | **Eigenvalue** | **Difference** | **Proportion** | **Cumulative** |
| --- | --- | --- | --- | --- |
| Factor1 | 10.149 | 7.686 | 0.677 | 0.677 |
| Factor 2 | 2.463 | 1.482 | 0.164 | 0.841 |
| Factor 3 | 0.981 | 0.474 | 0.065 | 0.906 |
| Factor 4 | 0.507 | 0.279 | 0.034 | 0.940 |
| Factor 5 | 0.228 | 0.022 | 0.015 | 0.955 |
| Factor 6 | 0.205 | 0.068 | 0.014 | 0.969 |
| Factor 7 | 0.138 | 0.037 | 0.009 | 0.978 |
| Factor 8 | 0.101 | 0.040 | 0.007 | 0.985 |
| Factor 9 | 0.061 | 0.009 | 0.004 | 0.989 |
| Factor 10 | 0.052 | 0.008 | 0.003 | 0.992 |
| Factor 11 | 0.044 | 0.012 | 0.003 | 0.995 |
| Factor 12 | 0.032 | 0.012 | 0.002 | 0.997 |
| Factor 13 | 0.020 | 0.008 | 0.001 | 0.999 |
| Factor 14 | 0.012 | 0.004 | 0.001 | 1.000 |
| Factor 15 | 0.008 | . | 0.001 | 1.000 |

**Table S3**

*Factor Loading Coefficients for the Three Principal Components*

| **Component** | **Variable** | **Factor1** | **Factor2** | **Factor3** | **Uniqueness** |
| --- | --- | --- | --- | --- | --- |
| Facilities levels | Activity room area | 0.975 | -0.081 | -0.111 | 0.031 |
|  | Toilet area | 0.978 | 0.007 | -0.097 | 0.034 |
|  | Sleeping room area | 0.938 | -0.090 | -0.125 | 0.097 |
|  | Health room area | 0.892 | -0.289 | -0.187 | 0.086 |
|  | Library area | 0.953 | -0.166 | -0.132 | 0.046 |
|  | Kitchen area | 0.952 | -0.015 | -0.109 | 0.081 |
|  | Green spaces area | 0.887 | 0.033 | -0.317 | 0.112 |
|  | Sports fields area | 0.887 | -0.187 | -0.226 | 0.128 |
|  | Number of books | 0.628 | 0.655 | -0.138 | 0.158 |
| Faculty levels | Number of teachers | 0.745 | -0.286 | 0.568 | 0.041 |
|  | Number of health practitioners | 0.719 | 0.563 | 0.233 | 0.112 |
|  | Number of caregivers | 0.203 | 0.880 | 0.160 | 0.159 |
|  | Number of directors | 0.653 | 0.645 | 0.187 | 0.123 |
| Number of kindergartens | Number of kindergartens | 0.680 | -0.510 | 0.448 | 0.076 |
|  | Number of kindergarten classes | 0.885 | -0.149 | 0.266 | 0.123 |
